# Multiple health-related behaviours among Fly-In Fly-Out workers in the mining industry in Australia: A cross-sectional survey during the COVID-19 pandemic

**DOI:** 10.1371/journal.pone.0275008

**Published:** 2022-10-27

**Authors:** Bernard Yeboah-Asiamah Asare, Elizabeth Thomas, Jacquita S. Affandi, Myles Schammer, Chris Harris, Dominika Kwasnicka, Daniel Powell, Christopher M. Reid, Suzanne Robinson

**Affiliations:** 1 Health Psychology Group, Institute of Applied Health Sciences, University of Aberdeen, Aberdeen, United Kingdom; 2 Curtin School of Population Health, Curtin University, Bentley, Australia; 3 Division of Surgery, School of Medicine, The University of Western Australia, Nedlands, Australia; 4 Mineral Resources Limited, Applecross, Australia; 5 Faculty of Psychology, SWPS University of Social Sciences and Humanities, Wroclaw, Poland; 6 NHMRC CRE in Digital Technology to Transform Chronic Disease Outcomes, Melbourne School of Population and Global Health, University of Melbourne, Melbourne, Australia; 7 Rowett Institute, University of Aberdeen, Aberdeen, United Kingdom; 8 School of Public Health and Preventive Medicine, Monash University, Melbourne, Australia; LSU Health Sciences Center New Orleans: Louisiana State University Health Sciences Center, UNITED STATES

## Abstract

**Background:**

Fly-In-Fly-Out (FIFO) workers travel to work at isolated locations, and rotate continuous workdays with leave periods at home, and such work practice is common in the offshore oil and gas and onshore mining industry worldwide. The COVID-19 pandemic and accompanying public health actions appear to have had a negative impact on several health-related behaviours among the general population. However, little is known about the impact of the COVID-19 pandemic on the health behaviours of FIFO workers, who have shown higher pre-pandemic rates of risky behaviours than the general population in Australia. This study examined the health-related behaviours of FIFO workers in the mining industry during the COVID-19 pandemic.

**Methods:**

A descriptive cross-sectional study was conducted. FIFO workers from an Australian mining company who underwent COVID-19 screening between May and November 2020 completed an online survey about their regular health-related behaviours. The independent sample t-test and Pearson’s chi-square test where appropriate were conducted to examine the differences between males and females for the behavioural outcomes.

**Results:**

A total of 768 FIFO workers (633 males and 135 females) participated in the study. Prevalence of smoking was high (32%). Males smoked more cigarettes per day than females (15.2±7.0 vs 13.1±7.1, *p* = .174). Most participants (74.7%) drank alcohol on more than two days per week. Compared to females, more males (20.2% vs 8.0%) consumed alcohol at short-term harmful levels (*p* = .010). About a third (34.4%) of the workers (33.5% of males and 38.5% of females, *p* = .264) engaged in inadequate moderate-vigorous exercises/physical activity. About a third (33.1%) of workers (33.7% of males and 30.4% of females; *p* = .699) had multiple risk behaviours.

**Conclusions:**

Prevalence of multiple risk behaviours was high. Interventions aimed at the prevention of risky health-related behaviours should target the different behavioural patterns and may require emphasis on gender-informed techniques particularly when addressing alcohol consumption.

## Introduction

Risky health-related behaviours such as tobacco use, lack of physical activity, and excessive alcohol intake are connected to a higher risk of premature death from non-communicable diseases (NCDs) [1, 2], particularly cardiovascular disease, cancer, respiratory disease, and diabetes [1]. The use of tobacco (including smoked, second-hand, and chewing) was estimated to cause 8·71 million deaths, constituting 15·4% of all mortalities worldwide in 2019 [2]. Similarly, in 2018 the use of alcohol at harmful levels was attributed to 5.3% of deaths globally [3]. In Australia, modifiable behaviour-related risk factors were attributed to about 38% of the burden of disease in 2018; including 8.6% attributed to tobacco use and 4.5% attributed to alcohol consumption [4].

Several risky health-related behaviours are indicated to frequently occur together [5, 6] and interrelate with one another [5, 7], commonly referred to as multiple risk behaviours, and observed in most adults [8]. Individuals who engage in multiple risk behaviours have a higher risk of developing chronic disease and dying young than those who engage in low-risk (zero to one) behaviours [9]. Reduction in modifiable behavioural risk factors is indicated to be a cost-effective strategy in the prevention and management of NCDs to reduce their negative consequences on individuals and society [1]. There is therefore the need to consistently observe the changes in the levels and trends of these behavioural risk factors in specific populations [8].

The novel Coronavirus (COVID-19) pandemic and accompanying public health and social restriction actions aimed at limiting the spread of the SARS-CoV-2 [10] have been shown to impact negatively on several health-related behaviours among the general population [11–14]. For instance, in the Australian general population, evidence has indicated a decline in physical activity/exercise compared to the pre-pandemic periods [14, 15], and a high proportion (30.8%) of alcohol consumers reported a lot more than the usual intake [16]. Furthermore, higher proportions of the general population have indicated increases in smoking since the COVID-19 pandemic began [15, 17]. Stressful events such as disease outbreaks/pandemics are associated with negative health-related behaviours [18], including high alcohol intake, used to cope with the associated stressors [19]. It has been shown that the experiences of increased distress, loneliness and hopelessness during the COVID-19 pandemic are associated with negative health behaviours, such as increased alcohol intake [20].

Disease outbreaks/pandemics are indicated to impact differently on women and men; with differences in exposure risk and genetic vulnerability to infection and socio-economic consequences, experiences during pandemics may differ in relation to people’s biological and gender characteristics [21]. Gender differences in health behaviors during the pandemic have been highlighted in the general adult population. Studies have shown women compared to men in the general population reported higher rates of reduced physical activity [22, 23] and increased the intake of junk food [24] during the COVID-19 pandemic. Other studies have also reported increased smoking and alcohol use among men compared to women [20, 22, 25–27] during the pandemic. Furthermore, a study established more women than men reported increases in the amount of food intake and weight and poorer sleep quality and duration during lockdown periods [28]. Evidence has indicated that women are more vulnerable to experiencing pandemic-induced psychological distress [29] and for instance, may use alcohol to cope with stress as compared to men [30]. It is as such suggested that studies assessing the health and socio-economic impact of the COVID-19 pandemic should include examining the gender disparities to inform appropriate responses [21].

Health-related behaviours have been linked to workplace outcomes including work injuries, sick leaves/absenteeism and loss of work productivity [31, 32]. Inadequate physical activity and smoking are associated with absenteeism due to sicknesses; smoking and insufficient fruit and vegetable consumption are associated with work productivity loss [31]. Increased alcohol intake are also linked to workplace injuries [33]. A high prevalence of risky health behaviours has been documented among workers in the resources industry in Australia [34, 35] and individuals working in this industry could be susceptible to these behaviours. The resources industry in Australia has for over two decades increasingly employed workers on Fly-In-Fly-Out (FIFO) arrangements, particularly in the mining sector on account of the remoteness of work sites, among other reasons [36]. FIFO workers are mostly flown to work at isolated locations, rotate continuous workdays with periods at home, typically on a compressed day and/or night shifts of 12 hours on average and live on-site in provided accommodation away from their families [37]. FIFO work arrangements are practiced in the offshore oil and gas and onshore mining industry around the work, particularly in Australia, Canada, Norway, and United Kingdom [38].

Previous pre-pandemic studies have highlighted FIFO workers in the resources industry have previously reported high levels of distress, poor sleep and risky health behaviours including higher smoking and alcohol use; and more overweight/obese compared to the general population [38] and compared with other employment types [39]. Evidence has indicated that those who consumed excessive alcohol prior to the pandemic are more likely to increase alcohol intake during the COVID-19 pandemic [16], suggesting that FIFO workers may be experiencing negative health behaviours for the period of the outbreak of the COVID-19 pandemic. Furthermore, the COVID-19 pandemic and accompanying control measures suggest further separation of workers from their close families and social support systems. FIFO workers may be subjected to travel quarantines as well as self-isolations and likely prolonged work periods in the course of COVID-19 lockdowns. These may further heighten the distress and loneliness seen in a number of workers and lead them to engage the risky behaviours [18, 19] such as smoking and alcohol use. However, little is known about the health behaviours of FIFO workers and the gender-related differences that may exist during the COVID-19 pandemic.

Knowledge about the gender differences and risky health-related behaviours could help in providing suitable and targeted support in FIFO workers during the COVID-19 pandemic and any subsequent stressful events. Gender-specific interventions have the potential to remove barriers caused due to gender bias that hinders the access to and uptake of interventions. For instance, it has been documented that interventions targeting women could reduce the obstacles to treatment entry and well serve the individual desires of smokers and have assisted women to quit smoking [40]. Gender focused intenventions are likely to target the vulnerable groups and address specific risk factors. For instance, considering gender analysis in identifying gender-specific risk factors has been indicated useful in the treatment and management of cardiovascular diseases [41].

Furthermore, knowledge about multiple risk behaviours and relationships between behaviours in FIFO workers would enable the design of effective and targeted interventions addressing single or multiple behaviours, for specific subgroups of FIFO workers, such as those who smoke cigarettes and drink and those who engage in insufficient physical activity [7].

This study focused on FIFO workers in the mining industry, examining the prevalence of risk behaviours and exploring the differences between men and women while investigating the correlations between common relevant health-impacting behaviours including tobacco smoking, alcohol consumption and physical activity critical in driving risks for chronic diseases burden [1] for the early period of the COVID-19 pandemic.

## Materials and methods

### Study design, participants and procedure

This study was a descriptive cross-sectional study among a self-selected convenient sample of FIFO workers of a large Western Australian mining company. All FIFO workers were invited to participate in the study during a COVID-19 screening program the mining company organised for its FIFO workers (N = 9,301) from May to November 2020. During the period, 1200 of the workers voluntarily opted to participate in the study and 768 (64%) of them responded by providing valid data to be included as the final sample for the study.

A structured online questionnaire, drawing on validated scales, e.g., [42, 43] and previous literature, e.g., [34, 44], was formulated by the authors and pre-tested. Participants anonymously completed the online questionnaire (set up in Redcap data capture tool), from May and November 2020. Participants’ recruitment and online data collection procedures have been described in detail elsewhere [45]. All participants provided an informed written consent before taking part in the study. The Curtin University Human Research Ethics Committee provided the ethics approval for this study (Approval reference number: HRE2020-0180).

### Measures

Participants provided information on socio-demographic characteristics (age, gender: participants identified as either male or female) and three health-related behaviours comprising alcohol intake, smoking, and physical activity since COVID-19 public health and social restrictions actions including working from home and social distancing started. Data collection was conducted between May to November 2020. In January 2020, the initial case of COVID-19 was recorded in Australia and by March 2020, various COVID-19 restrictions and lockdown measures were in place, up until May 2020 when restrictions began to ease [46]. The various COVID-19 related control measures including periodic lockdowns, social distancing, testing, mask wearing and quarantines have since continued.

#### Smoking behaviour

History of Smoking was measured using the item “Did you ever smoke tobacco regularly for 5 or more days a week for at least a year?” on a binomial scale (*Yes*: *smoker and No*: *non-smoker*). Current smoking status was assessed by asking participants to report on a binominal scale (*current daily smoker or ex-smoker)*. In addition, participants who smoked reported on the number of cigarettes usually smoked each day since COVID-19 public and social restrictions actions started.

#### Alcohol consumption

Participants reported on their history of alcohol intake on a 3-point scale (*current consumer*, *former consumer and never consumed alcohol*). Current alcohol consumption was assessed with modified items adopted from the AUDIT-C scale [42]. Participants reported on the item “How often do you usually drink alcohol since COVID-19 restrictions started?” on a 5-point scale (*Less than once a week*, *1–2 days per week*, *3–4 days per week*, *5–6 days per week*, *Everyday*). Participants also reported on the item “How many standard drinks would you usually have on a typical day when drinking since COVID-19 restrictions started” on a 5-point scale (*1–2 drinks*, *3–4 drinks*, *5–8 drinks*, *9–12 drinks*, *13 or more drinks*). Lifetime drinking risk was classified as drinking more than two standard drinks per day and short-time drinking risk was classified as consumption of more than 4 standard drinks per day [39].

#### Physical activity/exercise

Physical activity/exercise was assessed on undertaking moderate-to-vigorous activities since COVID-19 public restrictions started. Participants reported on the number of days per week they usually participate in moderate-to-vigorous-intensity activities. The Centre for Disease Control and Prevention (CDC) recommends 2 or more days a week of a combination of moderate- and vigorous-intensity activities to be of health benefits [47]. Physical activity days were stratified into two categories: inadequate moderate-vigorous physical activity (0–1 day) and adequate moderate-vigorous physical activity (2–7 days).

#### Multiple health-related behaviours

Multiple risk behaviours were measured on three common relevant health-related behaviours including alcohol intake, smoking and physical activity [1, 48]. A score of 1 each was given for engaging in current smoking, consuming more than 2 standard alcohol drinks per day and engaging in inadequate moderate-vigorous physical activities/exercises. A composite score ranging from 0–3 was created for participants by adding the total number of health-related behaviours for each participant. Participants were classified as engaging in no-risk behaviour (score 0), 1 risk behaviour (score 1) and multiple risk behaviours (scores 2–3) [48].

### Statistical analysis

The STATA version 13 (StataCorp LP, College Station, TX, USA) was used to conduct the data analysis. Continuous variables were presented in mean and standard deviation (±SD) and categorical variables in frequencies and proportions for descriptive purposes. The prevalence (with 95% confidence intervals) of smoking, alcohol consumption and physical activity outcomes for each gender (males and females) were identified. The independent sample t-test for continuous variables and the Pearson’s chi-square test (with post hoc analysis estimating the adjusted residuals and the corresponding p-values for each cell) for categorical variables were conducted to examine the differences between males and females for the behavioural outcomes. Spearman’s rank non-parametric correlations were conducted on health-related behaviours. *P*-value < .05 was considered statistically significant.

## Results

### Background characteristics of study participants

The participants were on average aged 44.1±11.8 (range 19–73) years; majority of them (74.7%) were aged more than 34 years. Most participants were of European descent (80.7%), male (82.4%) and the remainder were female (17.6%).

### Prevalence of health-related behaviours among participants

Table 1 presents the distribution of health-related behaviours across male and female FIFO workers. Thirty-two per cent of the participants had ever smoked tobacco regularly for 5 days or more for at least a year, with no significant differences between the males and females (32.9% vs 28.1%; χ^2^ = 1.1344, *p* = .287). Approximately 18% were current smokers, smoking on average 14.8±7.3 cigarettes per day since COVID-19 restrictions started. Although similar current smoker prevalence was found in women (19.3%) and men (17.4%) (χ^2^ = 0.2704, *p* = .603), male smokers were found to smoke more cigarettes per day than females (15.2±7.0 vs 13.1±7.1; t = 1.3684, *p* = .174).

**Table 1:** Lifestyle behaviours distribution across gender of FIFO workers.

Lifestyle behaviours	Total, %(95%CI) (n = 768)	Male, %(95%CI) (n = 633)	Female,%(95%CI) (n = 135)	χ2	*p*-value
**Ever smoked tobacco regularly**	32.0(28.8, 35.4)	32.9(29.3, 36.6)	28.1(21.2, 36.4)	1.1344	0.287
**Current smoking status**				0.2704	0.603
Current smokers	17.7(15.2, 20.6)	17.4(14.4, 20.3)	19.3(12.6, 25.9)		
Never/Ex-smoker	82.3(79.4, 84.8)	82.6(79.5, 85.4)	80.7(73.2, 86.6)		
**Current alcohol use**				4.4905	0.106
Current user	74.7(71.5, 77.8)	74.9(71.3, 78.1)	74.1(66.0, 80.8)		
Former user	13.4(11.2, 15.9)	14.2(11.7, 17.2)	9.6(5.7, 15.9)		
Never	11.8(9.7, 14.3)	10.9(8.7, 13.6)	16.3(11.0, 23.6)		
**Frequency of Alcohol intake**				4.6350	0.327
Less than once a week	27.7(24.3, 31.7)	26.6(22.8, 30.8)	34.0(25.4, 43.9)		
1–2 days per week	33.3(29.5, 37.2)	33.1(29.0, 37.5)	34.0(25.4, 43.9)		
3–4 days per week	26.8(23.3, 30.6)	27.2(23.4, 31.4)	25.0(17.4, 34.5)		
5–6 days per week	7.1(5.3, 9.6)	7.6(5.5, 10.4)	5.0(2.1, 11.5)		
Everyday	4.9(3.4, 7.0)	5.5(3.8, 7.9)	2.0(0.5, 7.7)		
**Number of standard drinks per day**				9.2774	0.010^**¥**^
1–2 drinks	38.3(34.4, 42.4)	36.5(32.3, 40.9)	47.0(37.4, 56.8)		
Long-term risky drinking	43.6(39.5, 47.7)	43.3(38.8, 47.8)	45.0(35.5, 54.9)		
Short-term risky drinking	18.1(15.2, 21.5)	20.2(16.9, 24.1)	8.0(4.0, 15.3)*		
**Engage in vigorous exercise**				1.2466	0.264
Inadequate	34.4(31.1, 37.8)	33.5(29.9, 37.3)	38.5(30.7, 47.0)		
Adequate	65.6(62.2, 68.9)	66.5(62.7, 70.1)	61.5(53.0, 69.3)		

^**¥**^significant at p<0.05;

*adjusted residual from post hoc = -2.377, p = 0.018

The majority of participants (74.7%) were current alcohol users, with no significant differences between males and females (75.9% vs 74.1%; χ^2^ = 4.4905, *p* = .106). About a third of the participants (33.3%) reported consuming alcohol for 1–2 days per week since COVID-19 restrictions started, with statistically similar proportions of males (33.1%) and females (34.0%) consuming alcohol for 1–2 days per week (χ^2^ = 4.6350, *p* = .327). Overall, 43.6% of the participants (43.3% males and 45.0% females) reported consuming alcohol at a rate of 3–4 standard drinks per day. More males than females indicated consuming more than 4 standard alcohol drinks (20.2% vs 8.0%) per day (χ^2^ = 9.2774, *p* = .010). A post hoc analysis showed significantly fewer females compared to males to consume more than 4 standard alcohol drinks (adjusted residual = -2.377, *p* = .018).

About one-third of the participants (34.4%) engaged in inadequate moderate-vigorous exercises/physical activity. Similar proportions of both males and females (33.5% vs 38.5%, χ^2^ = 1.2466, *p* = .264) reported engaging in inadequate moderate-vigorous exercises/physical activities.

### Multiple health-related behaviours and correlation between behaviours

The study found 6.6% of the workers (7.0% males and 5.2% females) indicated all three risky health-related behaviours (smoking, alcohol use and inadequate physical activity), 26.4% (26.7% males and 25.2% females) indicated two of the risky health-related behaviours, 39.7% (39.7% males and 40.0% females) reported only one, and 27.2% reported no-risk behaviours. Overall, about a third of the participants (33.1%) reported 2 or more risky health-related behaviours and there was no statistical difference between males and females reporting 2 or more risk behaviours (33.7% vs 30.4%; *p* = .699). Twelve per cent of the participants reported a combination of smoking and alcohol consumption at risky levels (more than two standard drinks per day) (Table 2). However, no correlation was found between the number of cigarettes smoked per day and the number of standard drinks intake per day, but the number of cigarettes smoked per day correlated with days of moderate-vigorous physical activity (r = -0.18, *p* = .009). In addition, there was no correction between the number of standard drinks intake per day and days of moderate-vigorous physical activity (Table 3).

**Table 2:** Combination of risk behaviours across gender.

health-related behaviours	Total, %(95%CI) (n = 768)	Male, %(95%CI) (n = 633)	Female,%(95%CI) (n = 135)	χ2	*p*-value
**Risk combination**				5.2993	0.623
No risk	27.2(24.2, 30.5)	26.7(23.4, 30.3)	29.6(22.5, 37.9)		
Physical inactive only	13.3(11.1, 15.9)	12.5(10.1, 15.3)	17.0(11.6, 24.4)		
Alcohol only	18.4(15.8, 21.3)	19.0(16.1, 22.2)	15.6(10.3, 22.7)		
Smoking only	8.1(6.3, 10.2)	8.2(6.3, 10.6)	7.4(4.0, 13.3)		
Alcohol and smoking	12.0(9.9, 14.5)	12.6(10.3, 15.5)	8.9(5.1, 15.0)		
Alcohol and physical inactive	9.1(7.3, 11.4)	9.0(7.0, 11.5)	9.6(5.7, 15.9)		
Smoking and physical inactive	5.3(4.0, 7.2)	5.1(3.6, 7.1)	6.7(3.5, 12.4)		
Alcohol, smoking and physical inactive	6.6(5.1, 8.6)	7.0(5.2, 9.2)	5.2(2.5, 10.5)		
**No of risk behaviours**				0.9736	0.808
No risk	27.2(24.2, 30.5)	26.7(23.4, 30.3)	29.6(22.5, 37.9)		
One risk	39.7(36.3, 43.2)	39.7(35.9, 43.5)	40.0(32.0, 48.5)		
Two risks	26.4(23.4, 29.8)	26.7(23.4, 30.3)	25.2(18.6, 33.2)		
Three risks	6.6(5.1, 8.6)	7.0(5.2, 9.2)	5.2(2.5, 10.5)		

**Table 3:** Correlations of health-related behaviours.

**Risk behaviours**	**Cigarettes per day**	**Standard alcohol drinks per day**	**Moderate-vigorous activity days per week**
**Cigarette per day**	1		
**Standard alcohol drinks per day**	0.04	1	
**Moderate-vigorous exercise days per week**	-0.18*	0.08	1

*p<0.05

## Discussion

### Main findings

This study sought to examine the prevalence of health-related behaviours, highlighting the gender differences, during the COVID-19 pandemic among FIFO workers in the mining industry. These common health-related behaviours comprising alcohol intake, smoking and physical activity, contribute substantially to morbidities and mortalities from NCDs [1]. The prevalence of current daily smoking (17.7%) among FIFO workers in our study was slightly higher than the 16% noted in a pre-pandemic study [44]. Our current finding was also higher than the pandemic rate of 10.7–13.4% [49, 50] and pre-pandemic rates (regular smokers: 13% and current smokers: 14.7%) [51] reported in the Australian adult general population. The current study found similar proportions of males and females to be current daily smokers, contrary to that established in the Australian adult general population where a higher rate of current daily smoking during the pandemic was reported in males (12.6%) than in females (8.8%) [52]. FIFO workers in the current study were found to smoke an average of 14.8 cigarettes per day, higher than the 13.2 cigarettes per day reported before the pandemic [53]. Our finding is also higher than the 10.7 cigarettes per day during the pandemic [52] and 12.9 cigarettes per day prior to the pandemic [36] reported among current smokers in the Australian general population. Male FIFO workers smoked more cigarettes per day than females, but difference was statistically insignificant, a trend consistent with that reported in the Australian general population, during the pandemic (men: 11.0, women: 10.4 cigarettes per day) [52] and pre-pandemic (men: 13.1, women: 12.9 cigarettes per day) [54]. An increase in smoking during the COVID-19 pandemic has been reported in the general population [15, 17], which have been linked to the stress caused by the COVID-19 pandemic [55]. Furthermore, FIFO workers are documented to report higher pre-pandemic levels of smoking than in the general population [38] and compared to other industries outside the resource sector [39]. Generally, shift workers report a high prevalence of smoking and a higher number of cigarettes smoked per day than non-shift workers [56]. FIFO workers work on compressed shift patterns of long hours [36], and rotation work in general is associated with high levels of psychological distress [38]. It has been widely documented that job stress increases the likelihood of smoking and the number of cigarettes smoked per day [57], as experiencing high levels of stress may increase the desire to smoke [58]. The COVID-19 pandemic and related preventive measures including restrictions have necessitated longer rosters, self-isolation/quarantine on-and-off sites and limited socialization, exacerbating further the experiences of distress among FIFO workers [59]. In our study, cigarette consumption reported in female FIFO workers were comparable to the pre-pandemic levels reported in the general population. However, studies examining smoking changes during COVID-19 pandemic have found females to be more likely to smoke more during the pandemic [17, 60], as a response to stressful events [30]. Evidence also suggests that females who work long hours, as done in the FIFO work arrangements, are found to be associated with increased levels of smoking [61].

Our study found high proportions of FIFO workers were current alcohol users (74.7%), with no significant differences between males and females. A high proportion of the participants (43.6%) reported consuming alcohol at a rate associated with long-term risk of harm, and about 18% of the workers consumed alcohol at a level exceeding the guideline for short-time risk of harm. While some studies did show an increase in levels of alcohol consumption during the pandemic in the adult general population in Australia [16, 62] with 25.8% of adults exceeding the *Australian Adult Alcohol Guideline* during the pandemic [63], previous studies have shown comparable higher consumption levels among FIFO and general mining workers during pre-pandemic periods [34, 39, 44]. This is supported by other pre-pandemic studies that have shown that mining workers consume alcohol at a higher rate than other professional industries [39], highlighting a need for further investigation into the cause of the behaviour that appears specific to the mining population [64].

More males (63.5%) than females (53.0%) indicated that they consume alcohol at risky levels. This finding is consistent with the findings made in the general Australian population during the pandemic where more men (33.6%) than women (18.5%) exceeded the national adult alcohol guideline [63]. However, other studies examining alcohol consumption during the pandemic have found females to consume more alcohol per week than males in the general Australian population [16, 65], with the female more likely to come under stress due to increased demands such as having to combine working at home and childcare as a result of lockdowns [16]. The observed differences could be accounted for by the differences in the study samples and measurements used. For instance, FIFO workers during work periods are free from household commitments such as childcare [66]. However, similar trends have been found in pre-pandemic studies among a mining population in Australia where more males (52.8%) than females (31.9%) reported risky or harmful use of alcohol [34, 35] and reported in the general population [67]. The FIFO workforce is mostly males [44] and on average, in the general population men consume more alcohol than women [68]. Furthermore, several other FIFO work characteristics including rotating shift patterns and shifts of more than 12 hours have been indicated to propagate the high and risky levels of alcohol consumption [35]. Additionally, FIFO campsites in Australia have been reported to not prohibit alcohol consumption as drinking is reported as a socialisation tool for workers at the worksite [69, 70]. On the other hand, FIFO workers are said to consume a lot of alcohol during their off-shift/leave periods [53], seen as a sign of freedom from the worksite which comes with some level or full restrictions [71].

Our study found about a third of the FIFO workers (34.4%) in this study engaged in inadequate moderate-vigorous exercises/physical activity (less than 2 or more day per week of moderate-vigorous exercises/physical activity), with no significant differences between male and female workers. This is lower than the 40.4% rate of insufficient physical activity reported among FIFO workers in the pre-pandemic periods [39]. The differences in periods between the studies and measures used should be noted; while the current study measured as the number of days per week they usually participate in moderate-to-vigorous-intensity activities, the previous study measured the ‘levels of physical activity during leisure time and work’. However, our current finding is in contrast to the findings of the National Health Survey among the Australian adult population during the pandemic where only 24.5% of adults meet the requirement for sufficient physical activity, with less women than men found to meet the physical activity guidelines (22.3% compared to 27.0%) [72]. Physical activities levels were generally indicated to have reduced in the general population due to COVID-19 restrictions around the world [73], including Australia [15, 74]. Previous studies have indicated a large proportion of FIFO workers engaged in leisure-time physical activities [38] in pre-pandemic periods as compared to other shift workers and work employment [39]. It has been suggested to be linked to the availability of satisfactory recreational facilities and, in some cases, fitness programs/activities for workers at worksites [75]. Further, owing to the fact that the usage of such facilities and participation in recreational activities is suggested to promote social interactions and a sense of belonging among workers [69]. However, COVID-19 restrictions and social distancing measures at both on-sites and at home may have limited social/recreational activities, including the social use of recreational facilities [59].

The study found a high prevalence (42%) of multiple (2 or more) health-related behaviours among both male and female FIFO workers. This is higher than reported in the Australian general population pre-pandemic, where the majority of people (51.1%) engage in one or more health-related behaviours, with 10.6% reporting two or more coexisting behaviours (smoking, alcohol intake and physical inactivity) and where males were more likely to engage in multiple risk behaviours [48]. This result may reflect the high proportions of FIFO workers reporting risky health-related behaviours than reported in the general population [38, 39] and the negative impact of COVID-19 impact of lifestyle behaviours [13, 15]. Differences in findings could also be attributed to the differences in measures of health-related behaviours across studies.

Our study found smoking was correlated with moderate-vigorous physical inactivity and not with alcohol consumption while physical inactivity was not correlated with alcohol consumption. These findings were partly supported by the findings among the Australian general population where physically inactive individuals were more likely to smoke whereas those in their 20s and 40s who smoked were more likely to consume alcohol at risky levels (more than 2 standard drinks per day) [48]. Again, dissimilarities in findings could be accounted for by the disparities in measures of health-related behaviours and the samples among the different studies. The findings of our study emphasise the need for interventions aimed at addressing or modifying health-related behaviours focusing on multiple behaviours, while taking into consideration relationships between behaviours, as against targeting a single behaviour, which is deemed to be more efficient to accomplish greater health benefits and cost-effectiveness [76].

### Strength and limitations

This study presents the gender differences in common health-related behaviours and multiple health behaviours among mining workers on FIFO work arrangements for the initial period of the outbreak of the COVID-19 in Australia. The study included relatively a large sample size. Nevertheless, some limitations to the study are noted. As a cross-sectional design study, where the time-based sequence cannot be determined, findings from this study cannot be interpreted with causality. There is the possibility that the prevalence and levels of health-related behaviours reported in the study could be under-or over-estimated because of the use of self-reported data. For instance, substance use is a sensitive social topic and there may also be strict workplace restrictions on their use, which could potentially lead to participants being reluctant to reveal their accurate substance use [34]. The levels of physical activity reported in this study may not accurately reflect the typical moderate/vigorous physical activity levels since the item used to assess physical activity did not identify the number of minutes spent per day engaging in physical activity. The study using convenient sampling could introduce self-selection biases and result in recruiting a sample that may not be representative of the general FIFO work population. Additionally, this study was descriptive and focused on examining the engagement of behaviours and the differences that may exist between men and women; however, other personal and workplace characteristics including FIFO job roles, shift pattern, shift hours, years spent working in FIFO roles could have influenced the health-related behaviours of FIFO workers [34, 35]. Further studies could examine the gender differences of workplace risks factors of behaviours to inform specific and targeted interventions to improve the lifestyle behaviours and health status of FIFO workers. The study included a relatively small number of females, which reflects the FIFO work population, predominantly includes males, and that could bias the observed differences in the study parameters. The study participants were reluctant to report on their educational level, marital status, and occupational roles, leading to several missing data and as such these variables were excluded, which limits the full description of the background characteristics of the study sample. The study included snap shot measures of health-related behaviours during the pandemic with comparisons made to the Australian norms and FIFO workers secondary data; assessing pre-pandemic health-related behaviours may enable for a more direct evaluations of the changes in the levels of behaviours during the pandemic.

## Conclusions

This study has indicated higher proportions of FIFO workers smoked cigarette, consumed alcohol at risky levels (short-to-long term risk) and engaged in sufficient moderate-vigorous physical activities/exercises during the COVID-19 pandemic compared to the Australian general population. However, the levels of behaviours may be comparable to pre-pandemic rates among FIFO workers. The prevalence of multiple risk behaviours was also high, and smoking was related to physical inactivity. Alcohol consumption was gender-related; the study indicated male FIFO workers consumed alcohol at risky levels more often than females. There is the indication for interventions aimed at the prevention or modifying of risky behaviours among FIFO workers to focus on multiple risk behaviours and their different patterns instead of focusing on single behaviours. Such interventions may also require emphasis on gender-informed techniques when addressing smoking and alcohol intake.
